# Supplementary material for: The SARS-CoV-2 Delta variant induces an antibody response largely focused on class 1 and 2 antibody epitopes
Source: PLoS Pathog. 2022 Jun 29;18(6):e1010592. doi: 10.1371/journal.ppat.1010592 (PMC9275729; doi:10.1371/journal.ppat.1010592)
Supplement: S1 Table — (DOCX) [file ppat.1010592.s001.docx]

**S1 Table. Summary characteristics of cohorts examined in this study**

| **Exposure history** | **Time period of sample collection** | **Location** | **Days post-symptom onset:** | **Number of individuals** | **Age** | **Number of females (%)** | **Study in which results first described** | **Types of data examined** |
| --- | --- | --- | --- | --- | --- | --- | --- | --- |
| Primary Delta infection | August– September, 2021 | South Africa | mean 33.4 (range 24–37) | 8 | Mean 47.4 (range 36–57) | 3/8 (37.5%) | Present study | Neutralization, DMS antibody-escape mapping |
| Delta breakthrough infection after 2x mRNA vaccination | July– September, 2021 | Washington State, USA | Mean 28.3 (range 24–38) | 8 | Mean 29.6 (range 20–68) | 5/8 (62.5%) | Present study | Neutralization, DMS antibody-escape mapping |
| Primary Beta infection | December 2020– January 2021 | South Africa | Mean 32.9 (range 27–40) | 9 | Mean 54.1 (range 26–78) | 5/9 (55.5%) | [[12]](https://paperpile.com/c/vSzLS9/qGzD) | Neutralization, DMS antibody-escape mapping |
| Early 2020 infection | Prior to March 15, 2020 | Washington State, USA | Mean 31.6 (range 15–61) | 17 | Mean 51.6 (range 23–76) | 8/17 (47.1%) | [[23]](https://paperpile.com/c/vSzLS9/XSEM) | Neutralization, DMS antibody-escape mapping |
| Early 2020 infection | Early 2020 | New York, USA [[65]](https://paperpile.com/c/vSzLS9/wMPB) | Mean 29 (range 21–35) | 5 | Mean 51.6 (range 42–66) | 2/5 (40%) | [[24]](https://paperpile.com/c/vSzLS9/toJk) | DMS antibody-escape mapping |
| 2x mRNA-1273* | Mid-2020 | USA (mRNA-1273 phase I trial [[66]](https://paperpile.com/c/vSzLS9/n5xx)) | 30 days post-dose 1 | 14 (after exclusion of one)* | Mean 31 (range recruited: 18–55) | 9/15 (60%) | [[29]](https://paperpile.com/c/vSzLS9/jGDl) | Neutralization, DMS antibody-escape mapping |
| 2x BNT162b2 | Early 2021 | Washington State, USA | Mean 31.2 days post-dose 1 (range 28–34) | 8 | Mean 34.5 (range 28–50) | 5/8 (62.5%) | Present study | Neutralization, DMS antibody-escape mapping |

*One participant was excluded from analysis due to not receiving a second vaccination dose but their demographic information is unknown.
