# Supplementary material for: The SARS-CoV-2 Delta variant induces an antibody response largely focused on class 1 and 2 antibody epitopes
Source: PLoS Pathog. 2022 Jun 29;18(6):e1010592. doi: 10.1371/journal.ppat.1010592 (PMC9275729; doi:10.1371/journal.ppat.1010592)
Supplement: S6 Fig — Delta mutant library escape maps for sera from individuals with (A) primary or breakthrough Delta infections after 2x mRNA vaccination (replicated here from Figs 3 and 5 to facilitate direct comparison) or (B) 2x BNT162b2 vaccination. Sites of strong antibody escape (see Methods) for any of the 8 plasmas in A or B are highlighted with pink in the line plots at left and shown in the logo plots at right. Sites 417, 452, 484, 477, 478, and 501 are included in the logo plots whether or not they are sites of strong escape due to their high frequency in circulating viral isolates. (C) The average site-total antibody escape for plasmas from individuals vaccinated with 2x mRNA-1273 against the Wuhan-Hu-1 mutant libraries (previously published in [29]) or 2x BNT162b2 against the Delta mutant libraries. Key epitopes are shaded. (D) The average site-total antibody escape mapped to the surface of the Wuhan-Hu-1 RBD (PDB 6M0J, [64]), with red indicating the site with the strongest antibody escape, and white indicating no escape. Key sites are labeled, with labels colored according to antibody epitope. The vaccine sera in B–D are from individuals who were not exposed to the Delta spike via infection or vaccination. The L452R mutation in the Delta RBD can disrupt antibody binding to both the class 2 and class 3 antibody epitopes [24,42], and thus the 2x BNT162b2 (x Delta mutant libraries) plasmas are mostly escaped by mutations in the class 4 epitope (including sites 365, 383, 384) or a non-canonical class 3 epitope that includes site 357. The antibody-escape maps against the Delta RBD mutant libraries are newly generated in this study, whereas the 2x mRNA-1273 antibody-escape maps against the Wuhan-Hu-1 RBD mutant libraries were first reported in [29] and are reanalyzed here. (PDF) [file ppat.1010592.s007.pdf]

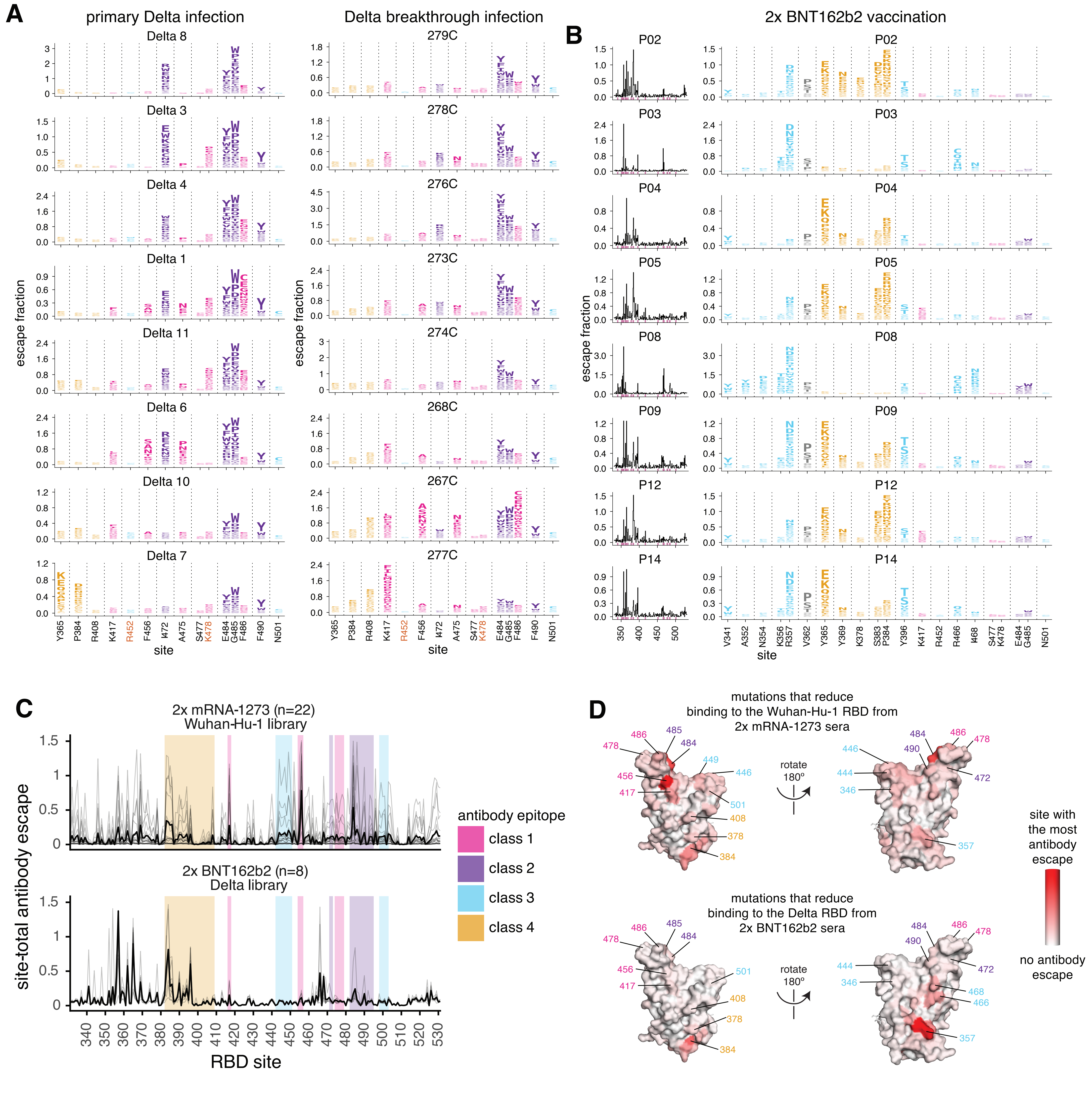

A
primary Delta infection
Delta 8
Delta 3
Delta 4
Delta 1
Delta 11
Delta 6
Delta 10
Delta 7
Delta breakthrough infection
279C
278C
276C
273C
274C
268C
267C
277C
escape fraction
Y365 P384 R408 K417 R452 F456 I472 A475 S477 K478 E484 G485 F486 F490 N501
site
B
2x BNT162b2 vaccination
P02
P03
P04
P05
P08
P09
P12
P14
escape fraction
350 400 450 500
V341 A352 N354 K356 R357 V362 Y365 Y369 K378 S383 P384 Y396 K417 R452 R466 I468 S477 K478 E484 G485 N501
site
C
2x mRNA-1273 (n=22)
Wuhan-Hu-1 library
2x BNT162b2 (n=8)
Delta library
site-total antibody escape
RBD site
340 350 360 370 380 390 400 410 420 430 440 450 460 470 480 490 500 510 520 530
antibody epitope
class 1
class 2
class 3
class 4
D
mutations that reduce
binding to the Wuhan-Hu-1 RBD from
2x mRNA-1273 sera
mutations that reduce
binding to the Delta RBD from
2x BNT162b2 sera
rotate
180º
site with
the most
antibody
escape
no antibody
escape
