# Supplementary material for: The SARS-CoV-2 Delta variant induces an antibody response largely focused on class 1 and 2 antibody epitopes
Source: PLoS Pathog. 2022 Jun 29;18(6):e1010592. doi: 10.1371/journal.ppat.1010592 (PMC9275729; doi:10.1371/journal.ppat.1010592)
Supplement: Attachment — Submitted filename: Delta_Reviewer_Responses.pdf [file ppat.1010592.s012.pdf]

Response to Reviewers

We thank you and the reviewers for their time and appreciation of our work. Please see the original reviewer comments in black, and our responses in blue.

Part I - Summary

Please use this section to discuss strengths/weaknesses of study, novelty/significance, general execution and scholarship.

Reviewer #1: The manuscript by Greaney et al. explores the variation of antibody responses following different types of infection. This is the latest in a series of manuscripts by the Bloom lab that uses their clever deep mutational scan approach to characterize antibody responses that the types of mutations that can evade them. The major conclusion is that Delta infections predominantly lead to type 1 and type 2 antibody classes. This is an excellent manuscript.

Thank you for the appreciation of our work.

Reviewer #2: Greaney and colleagues investigate the impact of differently SARS-CoV-2 exposure history on serum antibody specificity. This work directly compares serum from early pandemic infections, vaccinations, likely Beta infections, likely Delta infections, and delta breakthrough infections. They use deep mutational scanning to identify sites that disrupt serum antibody binding and find that exposure history influences the spectrum of “escape” mutations. The work is important, timely, expertly performed and well-written. I cannot offer meaningful improvements. Well done to all authors.

Thank you for the appreciation of our work.

Reviewer #3: This paper by Greaney et al builds on this team's well-established deep mutational scanning approach to show that infection by Delta triggers antibodies that differ subtly from those elicited by previous variants. Its elegant work and carefully described, though I note I am not able to evaluate the computational aspects of this study.

Thank you for the appreciation of our work.

Part II – Major Issues: Key Experiments Required for Acceptance

Please use this section to detail the key new experiments or modifications of existing experiments that should be absolutely required to validate study conclusions.

Reviewer #1: The one major comment is that as a reader I felt like there was a blaring omission. In the beginning and the end of the manuscript they show data from both infected, vaccinated,

and vaccinated breakthroughs. However, when they characterize the Delta responses in Figure 5, they only look at Delta primary infections and vaccinated with delta breakthroughs, but they don't show the data for vaccinated without a delta infection. It felt like a 2-legged stool. It's hard to believe they don't have this data. I think the authors really need to provide this data or at least provide the reader an explanation for why it is not included.

Thank you for raising this issue. We have previously mapped the antibody responses from mRNA-vaccinated individuals (https://www.science.org/doi/10.1126/scitranslmed.abi9915). We have now added these results to Fig 5 to facilitate comparison with Delta infection- and breakthrough-elicited responses.

Reviewer #2: (No Response)

Reviewer #3: None

Part III – Minor Issues: Editorial and Data Presentation Modifications

Please use this section for editorial suggestions as well as relatively minor modifications of existing data that would enhance clarity.

Reviewer #1: Minor comment. I could not find where the authors explicitly said how they decided what amino acid changes to include in their mutational maps (Fig 4B and 5A). I assume these were the most pronounced effects, but it would help to say so explicitly.

For each set of logo plos (Fig 3B, 5A, S6), sites of "strong escape" for any of the included plasmas or sera are highlighted in pink on the x-axis of the line plots, and featured in the corresponding logo plots at right. We define these sites of "strong escape" as those where the total escape (sum of mutation-level escape fractions) for that site exceeded the median across sites by > 10-fold, and was at least 10% of the maximum for any site. Sites 417, 452, 477, 478, 484, and 501 have been added to logo plots due to their polymorphism among circulating viruses.

We have now updated the Methods section, "Data visualization" with this additional information. Thank you for the helpful comment; we agree this improves the clarity and interpretation of results.

Reviewer #2: (No Response)

Reviewer #3: The title could be more informative in capturing variant specific nuances

We appreciate this suggestion, but were unable to come up with a sufficiently concise title that had more nuance. The current detail strikes the best balance we can come up with between accessibility / brevity and detail.

Figure 2 – it seems that though all responses are focussed on RBD, the slope of the curve varies in depletion experiments. Specifically Beta seems to have flatter curves. Is this meaningful ?

This may be partially due to the Beta plasmas having lower starting neutralization potencies, an effect that has been described by other groups (https://www.biorxiv.org/content/10.1101/2022.01.28.477987v1). The Beta plasmas do tend to have a lower fold-reduction in neutralization potency compared to the early 2020 or Delta primary infection plasmas, but this difference is not statistically significant. We do note, however, that this is a potentially intriguing trend, if indeed the anti-RBD response elicited by the Beta RBD is less potent than the anti-RBD response elicited by early 2020 or Delta viruses. It is tempting to speculate that this may be due to RBD mutations in major antibody epitopes.

Can the authors rule out prior infection? If not this should be mentioned in limitations

For all primary infection cohorts, individuals had no prior evidence of prior infection. This was corroborated by poorer binding and neutralization of plasmas against the non-homologous spikes and RBDs compared to that for the assumed spikes/RBDs of primary exposure. For example, all Delta primary infection plasmas had stronger binding and neutralization of Delta spike than early 2020 spike, whereas all Delta breakthrough samples had stronger binding and neutralization of early 2020 spike – which was included in the mRNA vaccines, the primary exposure – than to Delta spike.
